# Carcinoma de endometrio de alto riesgo en estadios iniciales: resultados oncológicos, Hospital Italiano de Buenos Aires

**DOI:** 10.31053/1853.0605.v80.n4.40821

**Published:** 2023-12-26

**Authors:** Diego Odetto, Maria C Puga, Guido M Rey Valzacchi, Jose M Saadi, Liliana B Zamora, Maria C Riggi, Myriam B Perrotta

**Affiliations:** 1 Sección Ginecología Oncológica, Hospital Italiano de Buenos Aires Argentina; 2 Sección Ginecología Oncológica, Hospital Italiano de Buenos Aires Ciudad Autónoma de Buenos Aires Argentina

**Keywords:** neoplasias de endometrio, sobrevida, endometrio, endometrial neoplasms, survival, endometrium, neoplasias do endométrio, sobrevida, endométrio

## Abstract

**Introducción:**

En Argentina el cáncer de endometrio es el segundo tumor ginecológico más frecuente, representando el 6% de todos los cánceres en mujeres. El objetivo de este trabajo es evaluar los resultados oncológicos y perioperatorios, en pacientes con cáncer de endometrio de alto riesgo (CEAR) limitados al útero tratadas en el Hospital Italiano de Buenos Aires de enero 2010-2018.

**Métodos:**

Estudio de cohorte retrospectivo que evaluó los resultados perioperatorios y la supervivencia libre de enfermedad entre los 2 y 4 años en pacientes con CEAR.

**Resultados:**

74 pacientes cumplieron con los criterios de inclusión. Los tumores serosos fueron los más frecuentes n=38 (51%), mientras que los desdiferenciados, los de menor frecuencia, n=2 (3%). 56 (76%) pacientes recibieron al menos un tratamiento adyuvante. El tratamiento sistémico fue implementado en 28 pacientes (38%), mientras que 24 (33%) recibieron una combinación de quimioterapia y radioterapia. La mediana de seguimiento fue de 2,9 años. La supervivencia libre de enfermedad, en pacientes con estadio IA a los 2 y 4 años fue de 71% (IC 95% 55-82) y 63 % (IC 46 -76) respectivamente, mientras que aquellas que presentaban un estadio IB fue de 53 (IC 95% 33-70) y 38 (IC 95% 19-58). En cuanto a la vía quirúrgica de abordaje, no se encontraron diferencias significativas en la supervivencia libre de enfermedad ni en las complicaciones perioperatorias.

**Conclusión:**

Sólo el estadio FIGO mostró un aumento en la probabilidad de muerte o recaída independientemente del tipo de tratamiento adyuvante realizado y de la vía de abordaje seleccionada.

Palabras clave: neoplasias de endometrio; sobrevida; endometrio

## Abstract (en)

**Background:**

Endometrial cancer is the second most frequent gynecological tumor in Argentina, representing 6% of all cancers in women. The objective of this study is to evaluate the oncological and perioperative results in patients with high-risk endometrial cancer (HREC) limited to the uterus, treated at the Hospital Italiano de Buenos Aires, between January 2010-2018.

**Methods:**

Retrospective cohort study that evaluated perioperative results, disease-free survival at 2, 4 years in patients with HREC.

**Results:**

Of a total of 123 patients, 74 met the inclusion criteria. Serous tumors were the most frequent histological type, n=38 (51%), while dedifferentiated tumors were the least frequent, n=2 (3%). Of all the patients included, 56 (76%) received at least one adjuvant treatment. Taxol platinum-based chemotherapy was implemented in 28 patients (38%), while 24 (33%) received a combination of chemotherapy and radiotherapy. The median follow-up time was 2.9 years. Disease-free survival in patients with stage IA at 2 and 4 years was 71% (95% CI 55-82) and 63% (CI 46-76), respectively, while those with stage IB were 53 (95% CI 33-70) and 38 (95% CI 19-58). Regarding the surgical approach, no significant differences were found in disease-free or overall survival when comparing the laparoscopic with the laparotomy approach (p=0.06).

**Conclusion:**

Only the FIGO stage showed an increased probability of death or relapse regardless of the type of adjuvant treatment and the type of surgery approach. Perioperative complications were similar in both approaches.

## Resumo

**Introdução:**

Na Argentina, o câncer de endométrio (CE) é o segundo tumor ginecológico mais frequente, representando 6% de todos os cânceres em mulheres. O objetivo deste trabalho é avaliar os resultados oncológicos e perioperatórios em pacientes com câncer de endométrio de alto risco (CHER) limitado ao útero, atendidas no Hospital Italiano de Buenos Aires, entre janeiro de 2010-2018.

**Métodos:**

Estudo de coorte retrospectivo que avaliou resultados perioperatórios, sobrevida livre de doença em 2, 4 anos em pacientes com HREC.

**Resultados:**

74 preencheram os critérios de inclusão. Os tumores serosos foram mais frequente, n=38 (51%), enquanto os tumores indiferenciados foram os menos frequentes, n=2 (3%). De todos os pacientes incluídos, 56 (76%) receberam pelo menos um tratamento adjuvante. A quimioterapia foi implementada em 28 pacientes (38%), enquanto 24 (33%) receberam uma combinação de quimioterapia e radioterapia. O tempo médio de seguimento foi de 2,9 anos. A sobrevida livre de doença em pacientes com estágio IA em 2 e 4 anos foi de 71% (IC 95% 55-82) e 63% (IC 46-76), respectivamente, enquanto aqueles com estágio IB foram de 53 (IC 95% 33- 70) e 38 (95% CI 19-58). Em relação à abordagem cirúrgica, não foram encontradas diferenças significativas na sobrevida livre de doença ou global ao comparar a abordagem laparoscópica com a laparotomia (p=0,06).

**Conclusão:**

Apenas o estágio FIGO apresentou maior probabilidade de óbito ou recidiva independente do tipo de tratamento adjuvante e do tipo de abordagem cirúrgica. As complicações perioperatórias foram semelhantes em ambas as abordagens.sincicial respiratório (VSR).

**CONCEPTOS CLAVE**

Que se sabe sobre el tema.

En Argentina, el cáncer de endometrio es el segundo tumor ginecológico más frecuente, representando el 6% de todos los cánceres en mujeres. Las pacientes pertenecientes al grupo de tumores de endometrio de alto riesgo (CEAR), tumores serosos, células claras, carcinosarcomas, y endometrioides poco diferenciados, presentan una supervivencia a los 5 años, que se encuentra entre 40-80% en la literatura mundial, pero desconocida en nuestro país.

Que aporta este trabajo.

Este trabajo propone como objetivo principal evaluar los resultados oncológicos y perioperatorios, en pacientes con carcinomas de endometrio de alto riesgo limitados al útero tratadas en un hospital de alto volumen en Argentina.

**Divulgación**

El cáncer de endometrio es un tumor frecuente en la población femenina Argentina. El pronóstico de estas pacientes se basa principalmente en la etapa en la cual se diagnostica la enfermedad y del subtipo histológico que presenta. Existen dos grupos de tumores, los de bajo riesgo que habitualmente se tratan con cirugía y presentan un pronóstico muy favorable y los de alto riesgo, con una incidencia menor, que también requieren de un tratamiento quirúrgico, pero a estos se les suma algún tipo de tratamiento extra, entre ellos radioterapia, quimioterapia o ambos. Este grupo de tumores tienen un pronóstico desfavorable, comparado al anterior. En nuestro país existe un déficit de información respecto a este subgrupo de tumores de endometrio de alto riesgo, por lo que nuestro trabajo propone aportar datos sobre la supervivencia de estas mujeres.

## Introducción

El cáncer de endometrio (CE) es el tumor maligno más frecuente del tracto genital femenino en países desarrollados, La tasa de incidencia estandarizada por edad varía de 1 a 30 casos por cada 100.000 mujeres en los países de todo el mundo ^1^ . En nuestro país, el cáncer de endometrio es el segundo tumor ginecológico más frecuente, representando el 6% de todos los cánceres en mujeres. Alrededor del 75% de las pacientes que lo padecen, presentan al momento del diagnóstico una enfermedad en estadio inicial, lo que la Federación Internacional de Ginecología y Obstetricia (FIGO) describe como estadio I o II, más específicamente, definido como enfermedad limitada al útero y sin signos de enfermedad a distancia ^2,3, 4^ .

Diferentes estudios demostraron que los tipos histológicos de bajo riesgo, tales como los adenocarcinomas endometrioides grado 1 y 2 presentan una supervivencia a 5 años cercana al 85% ^5^ . De modo contrario las pacientes pertenecientes al grupo de tumores de endometrio de alto riesgo (CEAR), tumores serosos, células claras, carcinosarcomas, y endometrioides poco diferenciados, presentan una supervivencia a los 5 años que va desde 40-80% ^6^ .

El tratamiento del CEAR depende principalmente de dos pilares, uno quirúrgico, al que se suma posteriormente, algún tipo de adyuvancia. Los tratamientos adyuvantes, suelen apoyarse en un régimen sistémico, basado en quimioterapia, un tratamiento local basado en radioterapia, e incluso, una combinación de ambos ^7^ .

Teniendo en cuenta que la supervivencia global a 5 años en estadios I-II, reportada por estudios multicéntricos ^6^ , se encuentra entre 40 a 80% resulta necesario conocer si en nuestra institución logramos obtener los mismos resultados. El objetivo principal del trabajo fue evaluar los resultados oncológicos y perioperatorios, en pacientes con carcinomas de endometrio de alto riesgo limitados al útero tratadas en el Hospital Italiano de Buenos Aires.

## Material y método

Estudio de cohorte retrospectivo que evaluó los resultados perioperatorios, la SLE a los 2 y 4 años y la supervivencia global en pacientes con CEAR, con enfermedad limitada al útero, tratadas en el Hospital Italiano de Buenos Aires (HIBA) entre enero del 2010 a junio 2018. El estudio fue aprobado por el Comité de Ética de Protocolos de Investigación del mismo hospital.

### Población

Se incluyeron pacientes con CEAR, tumores serosos, células claras, carcinosarcoma de endometrio, endometroides grado 3 y tumores desdiferenciados según la clasificación de la Organización Mundial de la Salud del 2020 ^8^ .Fueron incluidas pacientes mayores a 25 años, con tumores limitados al útero, estadios FIGO IA-IB-II, que recibieron el tratamiento de manera completa, es decir, cirugía, tratamiento adyuvante y seguimiento oncológico en nuestra institución. Se excluyeron aquellas pacientes operadas por recaída de su enfermedad, tratadas de manera primaria en otra institución, pacientes con tumores sincrónicos, o enfermedad oncológica activa con origen en otro órgano y pacientes con un tratamiento primario en nuestra institución pero con seguimiento en otro centro.

La decisión del tratamiento adyuvante se definió por un comité de tumores multidisciplinario. Las variables más importantes que se incluyeron en la evaluación fueron: edad, índice de masa corporal, imágenes preoperatorias, tipo de abordaje quirúrgico implementado, tiempo quirúrgico, estadía hospitalaria, complicaciones perioperatorias basadas en el score de Clavien Dindo ^9^ , performance status, estadio FIGO y tipo histológico.

### Análisis estadístico

Las variables continuas se describieron como media con su respectiva desviación estándar si poseían distribución normal, o como mediana si la distribución era asimétrica con sus respectivos rangos intercuartiles 25-75% (RIC) y se compararon mediante una prueba de T-de Student o una prueba de Mann-Whitney respectivamente. Las variables categóricas se reportaron como número o porcentaje y se compararon mediante prueba de Chi-cuadrado.

Se definió recaída como la presencia de enfermedad objetivable por imágenes o biopsia histológica luego de haber recibido un tratamiento primario. La SLE se definió como el tiempo transcurrido desde el tratamiento quirúrgico al diagnóstico de recaída o a la muerte. La SG se definió como el porcentaje de pacientes vivas hasta el último control del hospital o hasta el fin de seguimiento de la cohorte. El seguimiento luego de finalizado el tratamiento completo, incluyó una combinación de visitas clínicas cada 4 meses los primeros 2 años y cada 6 meses luego del 3º año. Se solicitaron estudios por imágenes en el seguimiento únicamente ante síntomas referidos por la paciente, o hallazgos en el examen clínico.

Para el análisis de la SLE y SG a los 2 y 4 años se utilizó el estimador de Kaplan-Meier. Las comparaciones entre los diferentes grupos se llevaron a cabo mediante el test de Mantel-Hazel.

Se construyó un modelo de riesgo proporcional de Cox para analizar la presencia de posibles confundidores en la estimación de SLE y de la SG. A los fines del análisis se re clasificó el estadío FIGO en 2 categorías (riesgo disminuido pacientes con FIGO IA y riesgo aumentado pacientes con FIGO IB y II). Los tratamientos adyuvantes, se agruparon en 2 categorías: pacientes con al menos un tratamiento y pacientes que nunca recibieron tratamiento luego de la cirugía. Se presentó su Hazard Ratio crudo y ajustado de cada predictor con su intervalo de confianza. Se testearon los supuestos de ausencia de correlación entre variables explicativas, homocedasticidad del modelo de regresión, proporcionalidad del riesgo. Los valores de p a dos colas inferiores a 0.05 se consideraron estadísticamente significativos. Para el análisis estadístico se utilizó el software STATA versión 13.

## Resultados

**Figura 1:**
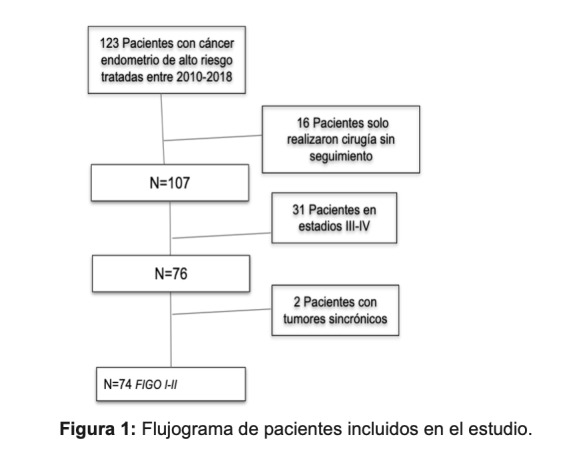
Flujograma de pacientes incluidos en el estudio.

Durante el periodo de estudio se identificaron 123 pacientes con CEAR estadio FIGO I-IV, de las cuales 49 fueron excluidas. (Figura 1) La cohorte quedó constituida por 74 pacientes cuyas principales características se describen en la Tabla 1.

**Tabla 1:** Características clínicas de las pacientes incluidas

Pacientes (n=74)	
Edad mediana en años (RIC)	67,5 (60-77)	
IMC mediana (RIC)	27 (24-35)	
Estadio FIGO, n(%)		
Bajo riesgo	42 (39,3)
IA	42 (39,3)
		
Alto riesgo IB	32 (43,24) 26 (24,3)
II		6 (5,6)
WHO Performance status n (%) 0 1 2	55 (75) 15 (20) 4 (5)

IMC: índice de masa corporal

En 66 pacientes (89%) la vía de abordaje fue por cirugía mínimamente invasiva, mientras que en 8 (11%) por laparotomía. La totalidad de las pacientes incluidas recibieron como tratamiento primario una anexohisterectomía total, a 56 de ellas (76%) se les realizó estadificación ganglionar pélvica y a 40 (54%) estadificación ganglionar pélvica y paraaórtica. Solo a 9 pacientes (12%) se las estadificó a través de la técnica de ganglio centinela, y a 25 pacientes (34%) una omentectomía completa. La mediana de tiempo quirúrgico fue de 180 minutos (RIC 150-240), y la de estadía hospitalaria de 2 días (RIC 2-3).

En relación a las complicaciones postoperatorias, se reportaron 7 incidentes (9%) de grado 3-4 según la clasificación de Clavien Dindo ^9^ . Una paciente presentó fibrilación auricular que requirió internación en una unidad cerrada, 3 pacientes intercurrieron con hematomas de cúpula vaginal que fueron drenados bajo anestesia, y dos pacientes presentaron una infección con colección pélvica que requirió una nueva intervención quirúrgica asociada a tratamiento antibiótico endovenoso. Una paciente de 78 años de edad falleció al día 32 de su internación. La muerte se produjo por una sepsis, a punto de partida de una lesión intestinal inadvertida en la cirugía primaria.

La mediana de ganglios pelvianos resecados fue de 15 (RIC 6 -17) y de ganglios lumboaórticos de 9 (RIC 3-12). Los tumores serosos fueron el tipo histológico más frecuente n=38 (52%), mientras que los desdiferenciados, los de menor frecuencia, n=2 (3%). Al analizar los tratamientos adyuvantes luego de la cirugía primaria se pudo evidenciar que del total de las pacientes incluidas 56 (76%) recibieron al menos un tratamiento adyuvante. En 28 pacientes (38%) se indicó quimioterapia basada en platino taxol, mientras que 24 (33%) recibieron una combinación de quimioterapia y radioterapia. En la tabla 2 se describen modalidades de tratamiento según estadio FIGO.

**Tabla 2:** Tratamientos adyuvantes según estadio FIGO

	IA (n=42)	IB (n=26)	II (n=6)
Tipo de tratamiento			
QMT+RT ∗, n (%)	18 (43)	4 (15)	2 (33)
RDT †, n(%)	13 (31)	12(46)	3 (50)
QMT ‡, n(%)	2 (5)	2 (8)	0 (0)
Ningún TTo §, n(%)	9 (21)	8 (31)	1 (17)

*- Quimioterapia sumado a radioterapia y/o braquiterapia.

†-Radioterapia pelviana sola con o sin braquiterapia.

‡- Quimioterapia como único tratamiento.

§-Ningún tratamiento adyuvante

Análisis de supervivencia libre de enfermedad (SLE) y supervivencia global (SG)

Durante el periodo de estudio el 36% (n=27) de las pacientes presentó una recaída (IC 95% 25-47%), siendo en el 78% (n=21) a distancia. De las recaídas locales 3/6 a nivel vaginal, y los 3 restantes a nivel pelviano. En este subgrupo de pacientes recaídas, 8/27 (30%) no pudieron recibir tratamiento adyuvante luego de la cirugía, debido a sus comorbilidades. El 97% (n=26) de las pacientes que sufrieron una recidiva, falleció durante el seguimiento, siendo la mediana de tiempo a la muerte de 2, 5 años (IC 95% 1,64-3,64).

La SLE a los 2 y 4 años en las pacientes se detalla en la tabla 3. La mortalidad cáncer específica fue del 35%, 11 pacientes murieron por alguna causa ajena a la enfermedad en estudio (ver anexo). La SG a los 2 y 4 años fue del 62% (IC 95% 50-72), 31 % (IC 95%18-46). En cuanto a la vía de abordaje, no se encontraron diferencias significativas en la SLE, ni en la SG, al comparar la vía laparoscópica con la vía convencional (p=0,57) (Figura 2).

**Tabla 3:** Supervivencia libre de enfermedad según estadio FIGO

	SLE 2 años - IC 95%	SLE 4 años - IC 95%	P valor
Figo IA	71% (55-82)	63% (46-76)	0,02*
Figo IB	53% (33-70)	38% (19-58)	>
Figo II	66% (19-90)	66% (19-90)	>

* p valor correspondiente a la comparación de la SLE según estadío

**Figure:**
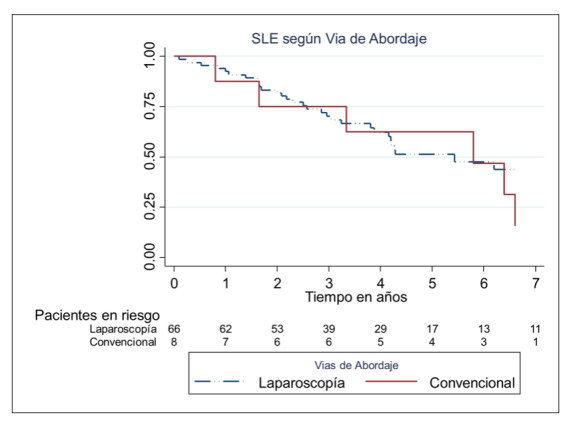

Al analizar la supervivencia libre de enfermedad según tratamiento adyuvante, se observó que en aquellas pacientes que realizaron un tratamiento combinado (radioterapia + quimioterapia) la SLE fue similar a los 2 y 4 años: 65% (IC 95% 42-80), mientras que en aquellas pacientes que recibieron sólo radioterapia, la SLE a los dos años fue de 63% (IC 95% 5-84) y a los 4 años de 57% (IC 95% 36-74). Finalmente, en las pacientes que no recibieron adyuvancia luego de la cirugía, la SLE a los 2 y 4 años fue de 38% (IC 95% 17-60) y 15% (IC 95% 2-38) respectivamente.

En el análisis multivariado de Cox el estadio FIGO mostró un aumento en la probabilidad de muerte o recaída independientemente del tipo de tratamiento y de la vía de abordaje. (Tabla 4)

**Tabla 4:** Regresión multivariada de Cox para SLE

	HR crudo (IC 95% )	p	HR ajustado (IC 95%)	p
FIGO Alto Riesgo	2,12 ( 1,10-4,0)	0,02	2,22 (1,14-4,30)	0,01
Tipo de Tto	1,03 (,53- 2)	0,92	1,20 (0.6-2.37)	0,63
Via de abordaje	1.46 (,61-3,51)	0,38	1,68 (1,68-2,42)	0,25

Tipo de Tto: Tipo de tratamiento adyuvante post operatorio.

## Discusión

En esta serie retrospectiva de un centro oncológicos integral de alto volumen, con cirujanos y oncólogos expertos en el tratamiento del cáncer de endometrio de alto riesgo, se pudo evidenciar un aceptable y bajo porcentaje de complicaciones perioperatorias graves tanto en la vía laparotómica, como en la minimamente invasiva. Luego del tratamiento quirúrgico 76% de las pacientes recibió al menos un tratamiento adyuvante, y solo el 33% recibió un tratamiento completo, es decir la combinación de la cirugía, con el tratamiento radiante y el sistémico. La SLE y SG fue similar en ambas vías de abordajes, siendo el principal factor pronóstico, el estadio FIGO al momento del diagnóstico y la adyuvancia recibida luego de la cirugía primaria.

Al evaluar las publicaciones más relevantes, observamos que en este subgrupo de pacientes con tumores de alto riesgo, aun en diferentes estadios FIGO, la combinación de tratamientos fue beneficiosa para las pacientes. En el ensayo ManGO ILIADE-III se mostró una mejora significativa en la supervivencia libre de enfermedad (SLE) a 5 años con quimio radioterapia versus radioterapia sola (78% vs 69%, p= 0,01) ^10^ . Por otra parte en el ensayo de fase 2 RTOG 9708, que investigó la radioterapia pelviana externa combinada con quimioterapia concurrente y adyuvante, reportó una supervivencia global (SG) a 5 años del 85% y una SLE del 79% ^11^ .

En el estudio PORTEC-3, ensayo aleatorizado más recientemente publicado, se informó una tasa SG estimada a 5 años del 79% y una supervivencia libre de enfermedad a 5 años del 73% en el grupo que recibió quimio radioterapia.

Para las mujeres con cáncer de endometrio en estadio I-II, el tratamiento adyuvante combinado produjo solo una pequeña mejoría absoluta del 2% (HR 0·84; IC del 95% 0·52–1·38) en la supervivencia general a 5 años y del 4% (0·87;0·56–1·36) en la supervivencia libre de enfermedad ^6^ .

En cuanto a los datos obtenidos en nuestro análisis, con una mediana de seguimiento de 2,9 años, ocurrieron 27 recaídas (36%) con una mediana de tiempo a la muerte luego de la recaída de 2,5 años (IC 95% 1,64-3,64). La SLE a los 2 y 4 años fue del 71 (IC 95% 55-82) y del 63% (IC 95% 46-76) respectivamente para los Estadios IA. En cuanto al estadio IB fue del 53% (IC 95% 33-70) y del 38 (IC 95% 19-58) respectivamente. Por último, en los estadios FIGO II, la SLE fue del 66% (IC 95% 19-90) a los 2 y 4 años respectivamente (p=0,02) **(Gráfico 1)**.

La supervivencia global de toda la cohorte a los 2 y 4 años fue del 62% (50-72), 31 % (18-46), respectivamente. De las 37 muertes reportadas durante el periodo analizado, 11 pacientes murieron por alguna causa ajena a la enfermedad en estudio.

Estos porcentajes de SLE y SG parecieran ser inferiores a los publicados en los ensayos clínicos anteriormente mencionados. Es importante destacar que en el análisis de nuestra cohorte, no se excluyeron pacientes que no recibieron ningún tipo de adyuvancia (N=18) o que solo hayan recibido braquiterapia, o únicamente radioterapia externa. Por otra parte, la mediana de edad de las pacientes tratadas en el PORTEC-3 fue de 62 años, mientras que en nuestra cohorte fue de 66 años, teniendo en cuenta que a más edad de la paciente, mayores comorbilidades presenta y menores chances de finalizar un tratamiento adyuvante tan prolongado, como el referido.

La evolución oncológica en términos de SLE, al analizar específicamente el tipo de tratamiento adyuvante efectuado, muestra los mejores resultados en el grupo que recibió quimioterapia y tratamiento radiante (la SLE a 2 y 4 años 65% (IC 95% 42-80) (gráfico 5), aunque un porcentaje menor, pero en concordancia con los 3 ensayos anteriormente mencionados ^6,10, 11^ .

Tanto en las guías del National Comprehensive Cancer Network (NCCN) (7) y en las de la sociedad Europea de Ginecología oncológica ^12^ , indican que la cirugía para este grupo de pacientes preferentemente debería hacerse por vía mínimamente invasiva. Hasta el momento, existen dos ensayos prospectivos aleatorizados que comparan la cirugía abierta vs la mini invasiva en pacientes con estadio temprano de cáncer de endometrio, no encontrando diferencias en términos de riesgo de recurrencia y supervivencia global ^13, 14^ . Ambos estudios se centraron en el cáncer de endometrio de bajo riesgo o tipo I (endometrioide) en lugar del tipo II (de alto riesgo) de cáncer de endometrio. Solo el 17,5% de los pacientes en el ensayo GOG LAP2 y un 6,5% en el ensayo LACE tenían tumores poco diferenciados.

Un trabajo reciente comparó los resultados obtenidos en pacientes con tumores de alto riesgo operadas por vía laparoscópica/robótica vs cirugía abierta. En este análisis no se encontraron diferencias significativas en complicaciones post-operatorias grado III (Clavien Dindo) entre ambos grupos (8,6% en el grupo de cirugía abierta vs 6,5% grupo de cirugía mínimamente invasiva) (p=0,32). Por otra parte, en el análisis de supervivencia, la cirugía mínimamente invasiva no se asoció a una peor SLE en comparación con la cirugía abierta [HR] 0,85; IC del 95%: 0,63-1,16; (p=0,30). La SLE a los 2 años fue del 71,6% (IC 95% 51,5-66,4) en el grupo de cirugía mínimamente invasiva y 69,2% (IC 95% 61,8-75,5) en el grupo convencional. En términos SG tampoco se asoció a la cirugía laparoscópica con peores resultados que a la cirugía abierta. (HR 1,04, IC del 95% 0,73–1,48, (p=0,81). La SG en el grupo mini invasivo vs cirugía convencional a 2 años fue de (85,8% [IC 95% 79,9-90,1 vs 86,3% [IC 95% 80,5–90,6], p=0,72) y a 5 años del 68,6% [IC 95% 61,1–74,9] frente al 72,2% [IC del 95%: 65,0 a 78,2], p=0,45) respectivamente ^15^ .

En nuestro estudio se pudo comprobar que la vía de abordaje mínimamente invasiva, parece ser una modalidad apropiada para tratar este subgrupo de tumores de alto riesgo. En nuestra cohorte el 89% (n=66) de las pacientes fueron operadas por vía laparoscópica, con una tasa de complicaciones de grado 3-4 según la clasificación de Clavien Dindo ^9^ del 9%, muy cercano al porcentaje reportado por el grupo del MD Anderson en asociación al grupo de la Mayo Clinic, citado recientemente ^15^ .

Como debilidades en este estudio, la naturaleza retrospectiva del mismo, es la principal limitación del trabajo. Este hecho limitó las variables seleccionadas para el análisis, aunque no alteró el objetivo primario que fue evaluar la SLE y SG. A los fines del análisis para evaluar la presencia de confundidores, se debió re clasificar el Estadío FIGO en 2 categorías (riesgo disminuido pacientes con FIGO I y riesgo aumentado pacientes con FIGO IB y II). Los tratamientos adyuvantes, se agruparon en 2 categorías: pacientes con al menos un tratamiento y pacientes que nunca recibieron tratamiento luego de la cirugía. No se logró obtener un número de pacientes similares para cada estadio, por lo que la SLE Y SG de pacientes con estadio II (n=6) no pareciera ser la real (63% IC 95% 16-86). Asimismo, tampoco se consiguió obtener una cantidad de pacientes considerable, para comparar la vía de abordaje mínimamente invasiva vs la convencional.

Las principales fortalezas de este estudio se basan en el número importante de pacientes con subtipos histológicos de baja incidencia como son los tumores de endometrio de alto riesgo, pertenecientes a un solo centro oncológico. Por otra parte, tanto el tratamiento quirúrgico, como el sistémico y el radiante fue realizado por médicos expertos, todos pertenecientes a un grupo multidisciplinario quienes desarrollan su principal actividad laboral en el Hospital Italiano de Buenos Aires. Cada etapa recorrida por las pacientes fue plasmada en una historia clínica electrónica única, desde el momento del diagnóstico y hasta el final del seguimiento. Por último, es el primer estudio en Argentina que combina los resultados perioperatorios de dos diferentes vías de abordajes, sumado a resultados de sobrevida libre de enfermedad y global en pacientes con tumores de endometrio de alto riesgo.

## Conclusión

En nuestra cohorte de pacientes solo el estadio FIGO mostró un aumento en la probabilidad de muerte o recaída independientemente del tipo de tratamiento adyuvante realizado y de la vía de abordaje seleccionada. Las complicaciones perioperatorias fueron similares en ambas vías de abordaje, aunque vale la pena destacar el menor tiempo de estadía hospitalaria y recuperación que tiene la cirugía laparoscópica. La vía de abordaje no pareciera ser un factor que modifique los resultados de sobrevida, aunque no existan aún ensayos clínicos prospectivos elaborados únicamente con CEAR que lo demuestren.
